# Effect of adhesive layers on microshear bond strength of nanocomposite resin to dentin

**DOI:** 10.4317/jced.53133

**Published:** 2017-02-01

**Authors:** Nayef H. Felemban, Mohamed I. Ebrahim

**Affiliations:** 1 Faculty of Dentistry, Taif University, Al Huwaya, Taif, Saudi Arabia

## Abstract

**Background:**

Bond strength of adhesive layer can absorb unwanted stresses of polymerization shrinkage in composite resin restorations; increased microshear bond strength can prevent failure of restoration materials, the purpose of this study was to evaluate the effect of adhesive layers on microshear bond strength of nanocomposite resin to dentin.

**Material and Methods:**

Two different types of adhesive systems: universal adhesive (ExciTE) and newly developed adhesive (Nano-Bond), and one type of light-cured resin restorative material (Nanocomposite resin) were used in this study. The occlusal surfaces of extracted human molar teeth were ground perpendicular to the long axis of each tooth to expose a flat dentin surface. The adhesives were applied on dentin surfaces (single application or double application). Nanocomposite resin was then placed and light cured for 40 seconds. After 24 hours of immersion in water at 37°C, then subjected to thermocycling before testing, a microshear bond test was carried out. The data were analyzed by a two-way ANOVA. For comparison between groups, Tukey’s post-hoc test was used.

**Results:**

The mean bond strengths of ExciTE and Nano-Bond adhesives with a single application were 8.8 and 16.6 MPa, respectively. The mean bond strengths of ExciTE and Nano-Bond adhesives with double application were 13.2 and 21.8MPa, respectively. There were no statistically significant differences in microshear bond strengths between the single application of Nano-Bond and the double application of ExciTE adhesives.

**Conclusions:**

Microshear bond strength increased significantly as the applied adhesive layer was doubled.

** Key words:**Adhesive, microshear, bond, strength, nanocomposite.

## Introduction

Stress arising from polymerization shrinkage is one of the most critical problems with light-activated composites (1). The competition between contracting forces built up in the polymerizing resin and the bonds of adhesive resins to the wall of restorations can lead to marginal failure and subsequent microleakage. For this reason, bond strength must be greater than contraction stress in order to obtain stable marginal adaptation (2,3).

From restorative dentistry, the use of bonding agents is known to improve the adhesion of composite resins. The bonding agents create a micromechanical interlock between the dentin collagen and resin by forming the hybrid layers (4).

The adhesives could be unfilled or filled ones. The unfilled adhesives have lower mechanical properties, which could lead to gap formation or recurrent caries at the margin of restoration. So the idea of filled adhesives has been introduced, (5) which includes various types of fillers, such as conventional glass, ion leachable glass, silica, and nano-sized aerosol silica filler (6,7). They have been reported to improve the marginal and internal seal of composite restorations (8).

Nano-sized filler was introduced to bonding agents by mean of nanotechnology. Nanofillers technology has been known to increase adhesion to both enamel and dentin and improves marginal integrity (9).

Investigators reported that the application of two coats (double-application) of the all-in-one bonding system successfully increased the bond strength to sound dentin (10). Morphological observation of the resin-dentin interface using the transmission electron microscopy (TEM) technique revealed that bonding an all-in-one adhesive to dentin was improved by the application of a second adhesive layer after light curing the first layer (11). Multiple consecutive coatings of another bonding system, the one-bottle total-etch adhesive system, also improved its bond strength and reduced nanoleakage (12). The current trends in bonding appear to favor single application of adhesives; however, it is speculated that the double-application method is an effective technique to improve bonding to dentin.

The purpose of this study was to evaluate the effect of the adhesive layers on microshear bond strength of nanocomposite resin to dentin. The null hypothesis was that the bond strengths of ExciTE and Nano-Bond adhesives are significantly different between single- and double-application methods.

## Material and Methods

Two different types of available adhesive systems, ExciTE (Ivoclar, Vivadent, Schaan, Liechtenstein, Swiss, lot #K41832) and Nano-Bond adhesives (Pentron Clinical Technologies, USA, lot #183421), and one type of nanofilled composite (Artiste Nano-composite, Pentron Clinical Technologies LLC, USA, lot #182066-185215) were used in this study. ExciTE is a fourth-generation universal adhesive system and Nano-Bond is fourth-generation newly developed adhesive system.

Twenty caries-free freshly extracted human molar teeth were collected for use in this study. The teeth were cleaned by ultrasonic scalers and stored in distilled water at 37°C before testing. A dentin slice, approximately 1.0mm thick, was cut perpendicular to the long axis of each tooth from the upper-middle coronal portion region using a low-speed diamond saw (IsoMet®, Buehler, Lake Bluff, IL) under water coolant. The occlusal surfaces of slices were ground with silicon carbide paper up to #600 grit to expose a flat dentin surface (13-16).

The dentin slices were divided into two main groups (containing 10 each) according to the type of adhesive system. Group A: ExciTE adhesive and Group B: Nano-Bond adhesive system. Each group was further subdivided into two subgroups (containing 5 each) according to layers of adhesive system. Subgroup 1 (A1 & B1): one layer of adhesive systems, and Subgroup 2 (A2 & B2): two layers of adhesive systems.

Each dentin slice was acid etched using 37% phosphoric acid gel for 15 seconds. Then the dentin slices were rinsed with water spray and dried with an oil-free stream for five seconds. Primer was applied on the etched dentin surfaces by using the applicator brush. The excess primer was removed with a dry applicator brush, but the surface had a very wet appearance. Then they were light cured for 10 seconds with light emitting diodes (LED). The adhesives were applied on the dentin surfaces by the appropriate manufactures’ instructions or by an experimental method (single or double application). The adhesives were applied to the entire dentin surface and air-thinned for 15 seconds. A gentle stream of dry air was applied to disperse the material into a thin, uniform, shiny surface and, prior to irradiation, three or four cylinders (internal diameter: 0.7mm, height: 1.0mm) of Tygon® microbore tubing (R-3603, Norton Performance Plastic Co., Cleveland, OH) were placed on the flat dentin at different locations. The adhesive was then light-cured for 10 seconds with LED. Specimens with thick adhesive layers were produced by the application of one additional coat of adhesive. Each layer was light-cured separately for 10 seconds.

After irradiation, each tube was filled with nanofilled composite resin and then light-cured for 40 seconds with the tip as close to the surface as possible. Curing radiometer equipment was used to ensure steady light intensity throughout the polymerization of all specimens. All restorations were finished and polished with a set of solfex discs (3M Company, St. Paul MN, USA). The specimens were stored in moist conditions at a room temperature of 23°C for one hour prior to removing the tygon tubing.

The specimens were immersed in water at 37°C for 24 hours then subjected to thermocycling to simulate clinical thermal stress conditions before testing, according to the American National Standards Institute/American Dental Association (ANSI/ADA) (17) and the International Organization for Standardization (ISO) (18) for direct filling resins and dental adhesions. All specimens were subjected to thermocycling by alternatively storing in water reservoirs at 5°C and 55°C, respectively, remaining in each reservoir for 30 seconds. This procedure was carried out for 500 cycles and was controlled by a computer to simulate thermal stress (19).

The resin cylinders were then subjected to the microshear bond test (15). Each dentin slice with the resin cylinders was placed in the lower attachment of the universal testing machine (model LRX-Plus II; Lloyd Instruments Ltd., Fareham, UK) for microshear bond testing. A thin wire (diameter 0.20mm) was looped around each resin cylinder, making contact through half of the cylinder base and was placed as close as possible to the resin-dentin interface. A shear force was applied to each specimen at a crosshead speed of 0.5mm/min until failure occurred. The resin-dentin interface of the specimens and the wire loop were aligned as straight as possible to ensure that the same orientation in shear was maintained. The loads at failure were recorded and the data were analyzed by a two-way ANOVA. Tukey’s post-hoc test was used for pairwise comparisons between the means when the ANOVA test was significant. the study has been approved by an Ethics Committee.

## Results

The mean percentage for the tested adhesives (ExciTE and Nano-Bond) with different interactions is presented in  Table 1. The Nano-Bond adhesive with two layers (B2) showed the statistically significant highest mean microshear bond strength (MPa). This was followed by Nano-Bond adhesive applied in one layer (B1), then of ExciTE adhesive applied in two layers (A2) with no statistically significant difference between the two interactions. The statistically significant lowest-mean microshear bond strength (MPa) was found with ExciTE adhesive applied in one layer (A1). The results of the microshear bond strength showed significant difference (*P*<0.05) between group B2 and group A1. Microshear bond strength was increased for the specimens that received two layers.

**Table 1:**
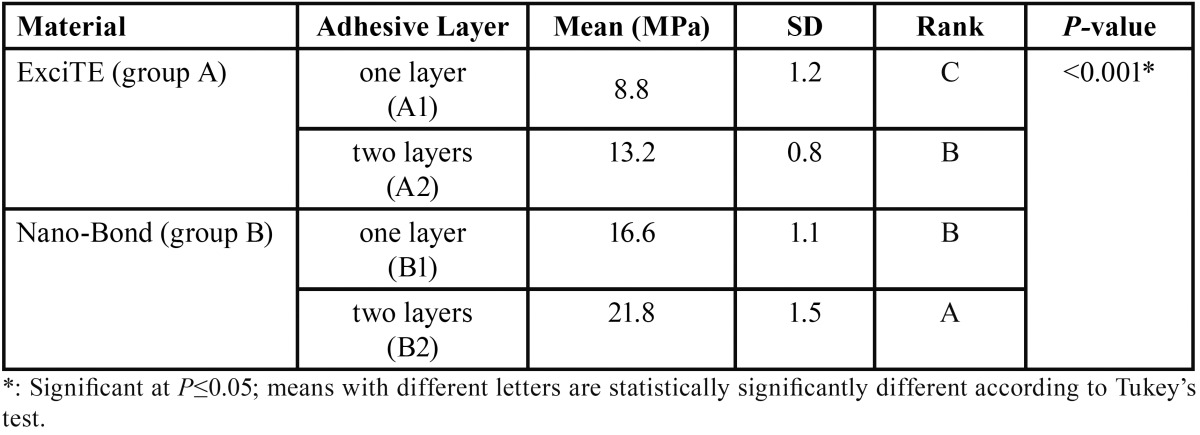
Comparison between microshear bond strength (MPa) of the tested adhesives with different interactions.

## Discussion

Dentin adhesives are currently available as three-step, two-step, and single-step systems, depending on how the three cardinal steps of etching, priming, and bonding-to-tooth substrates are accompanied or simplified. Three-step systems involve etching with an acidic condition, priming with hydrophilic resin in solvent, and bonding with an unfilled or lightly filled resin (20).

The major goals of using dentin bonding systems are to enhance the bonding strength between the resin and the tooth structure, increase the retention of restoration, reduce the microleakage across resin-dentin interfaces, and scatter the occlusal stress (21).

An *in-vitro* mechanical test became of utmost importance to evaluate and compare bond strengths of adhesive systems to enamel and dentin. The most commonly employed test setup for this purpose were tensile and shear tests (22,23). Shear bond strength tests have been widely used, mainly because of their relative simplicity when compared to tensile bond strength tests, in which it is difficult to align the specimen in the testing machine without creating deleterious stress distribution (24,25). Advantages in shear tests include specimen preparations and simple test protocols (13).

A new test method using specimens with reduced dimensions has been advocated by some authors (13,16,26,27) as a substitute for the conventional shear test: so-called microbond or microshear bond strength test. According to them, this test would allow for testing of small areas, thus permitting a regional mapping or depth profiling of different substrates and preparing multiple specimens from the same tooth.

Adhesive layers act as an elastic intermediate layer (elastic cavity wall) between cavity walls and the adjacent composite. This layer could resist the polymerization shrinkage stress of the resin composites and absorb the shock produced by occlusal loads and thermal cycling (28).

According to many investigators (5,9) the use of filled adhesive resin increases the mechanical properties and improves marginal and internal seals of composite restoration.

Adhesive systems have the acidic monomer ingredient, which demineralizes the subsurface of the dentin, removes or modifies the smear layer, and improves the infiltration of the adhesive resin through residual smear layers into underlaying dentin (11). These adhesives may be categorized as mild or strong adhesives, depending on their pH levels and, therefore, their etching potential (28). If the adhesive’s capacity to dissolve the smear layer is limited, the bond strength to dentin with a thick smear layer may be reduced (29).

Multiple applications of adhesives are considered to be effective to prevent the above-mentioned defects of resin bonding and improve the bond performance to dentin; (11,12) therefore, the effect of the double application of adhesive systems on the bond strength and quality of the resin-dentin interface is of crucial interest.

In this study, microshear bond strength of ExciTE and Nano-Bond adhesives showed a significant difference between single and double layers (*P*>0.05). The bond strength of ExciTE and Nano-Bond adhesives were increased with a double application as compared with that of a single application. Nara *et al.* (10) reported that two coats (a double application) of an all-in-one adhesive could increase the bond strength to that of sound dentin. On the other hand, when observing the fractured surfaces, the morphology of the intertubular dentin greatly varied between the two application methods. The single application produced a porous and fibrous appearance, which is supposed to be over-etched. In a double application, the intertubular dentin appeared to be dense. Single applications of the adhesive had the effect of a strong etchant, although the infiltration of resin to demineralize dentin may not be sufficient. In the second application, the additional supply of adhesive resin may improve the infiltration of resin monomer into the intertubular demineralized dentin (30).

## Conclusions

In this study, Nano-Bond adhesive showed greater microshear bond strength than that of the ExciTE adhesive. This may be contributed to the lightly nanofilled and decreased pH of the Nano-Bond adhesive. Low pH will greatly influence the demoralized degree of tooth substrate. So, the etching of Nano-Bond adhesive is strong enough to completely remove the dentin smear layer. This leads to sufficient infiltration of resin demineralized dentin and increases the bond strength.
